# Voice-Based Conversational Agents for the Prevention and Management of Chronic and Mental Health Conditions: Systematic Literature Review

**DOI:** 10.2196/25933

**Published:** 2021-03-29

**Authors:** Caterina Bérubé, Theresa Schachner, Roman Keller, Elgar Fleisch, Florian v Wangenheim, Filipe Barata, Tobias Kowatsch

**Affiliations:** 1 Center for Digital Health Interventions Department of Management, Technology, and Economics ETH Zurich Zurich Switzerland; 2 Future Health Technologies Programme Campus for Research Excellence and Technological Enterprise (CREATE) Singapore-ETH Centre Singapore Singapore; 3 Center for Digital Health Interventions, Institute of Technology Management University of St. Gallen St. Gallen Switzerland; 4 Saw Swee Hock School of Public Health National University of Singapore Singapore Singapore

**Keywords:** voice, speech, delivery of health care, noncommunicable diseases, conversational agents, mobile phone, smart speaker, monitoring, support, chronic disease, mental health, systematic literature review

## Abstract

**Background:**

Chronic and mental health conditions are increasingly prevalent worldwide. As devices in our everyday lives offer more and more voice-based self-service, voice-based conversational agents (VCAs) have the potential to support the prevention and management of these conditions in a scalable manner. However, evidence on VCAs dedicated to the prevention and management of chronic and mental health conditions is unclear.

**Objective:**

This study provides a better understanding of the current methods used in the evaluation of health interventions for the prevention and management of chronic and mental health conditions delivered through VCAs.

**Methods:**

We conducted a systematic literature review using PubMed MEDLINE, Embase, PsycINFO, Scopus, and Web of Science databases. We included primary research involving the prevention or management of chronic or mental health conditions through a VCA and reporting an empirical evaluation of the system either in terms of system accuracy, technology acceptance, or both. A total of 2 independent reviewers conducted the screening and data extraction, and agreement between them was measured using Cohen kappa. A narrative approach was used to synthesize the selected records.

**Results:**

Of 7170 prescreened papers, 12 met the inclusion criteria. All studies were nonexperimental. The VCAs provided behavioral support (n=5), health monitoring services (n=3), or both (n=4). The interventions were delivered via smartphones (n=5), tablets (n=2), or smart speakers (n=3). In 2 cases, no device was specified. A total of 3 VCAs targeted cancer, whereas 2 VCAs targeted diabetes and heart failure. The other VCAs targeted hearing impairment, asthma, Parkinson disease, dementia, autism, intellectual disability, and depression. The majority of the studies (n=7) assessed technology acceptance, but only few studies (n=3) used validated instruments. Half of the studies (n=6) reported either performance measures on speech recognition or on the ability of VCAs to respond to health-related queries. Only a minority of the studies (n=2) reported behavioral measures or a measure of attitudes toward intervention-targeted health behavior. Moreover, only a minority of studies (n=4) reported controlling for participants’ previous experience with technology. Finally, risk bias varied markedly.

**Conclusions:**

The heterogeneity in the methods, the limited number of studies identified, and the high risk of bias show that research on VCAs for chronic and mental health conditions is still in its infancy. Although the results of system accuracy and technology acceptance are encouraging, there is still a need to establish more conclusive evidence on the efficacy of VCAs for the prevention and management of chronic and mental health conditions, both in absolute terms and in comparison with standard health care.

## Introduction

### Background

Chronic and mental health conditions are increasingly prevalent worldwide. According to the World Health Statistics of 2020, noncommunicable diseases (eg, cardiovascular diseases, cancer, chronic respiratory diseases, and diabetes) and suicide are still the predominant causes of death in 2016 [1,2]. Although the underlying causes of these conditions are complex, behavior remains an important factor in their prevention and management. As the health care system is currently unfit to sustain the prevention and management of chronic and mental health conditions while containing its costs, continuous and personalized smartphone-based interventions have been developed to provide scaled-up behavioral support [3-6]. On the same note, conversational agents have been proven a valuable tool to deliver digital health interventions [7-9]. In particular, voice-based conversational agents (VCAs) have been shown to provide high user satisfaction in delivering interventions to influence healthy lifestyles [6].

VCAs can recognize human speech and, in turn, respond with synthesized speech. The human input is converted into an intent, triggering a specific information retrieval or function. This modality of interaction allows for hands-free access to some basic functions, such as searching for information on the internet, managing calendars, playing media content, calling, texting, emails, controlling internet-of-things devices and telling jokes [10,11]. Just as text-based [12,13] and embodied [14] conversational agents, VCAs have the potential to form an *alliance* [15] or *rapport* [16] with the patient through conversation, which is beneficial to treatment outcomes [17-19]. Compared with text-based interactions, however, voice-based interactions have several advantages. First, voice-based interaction leverages the naturalness [20,21] and social presence [22,23] of human-to-human conversation. Second, it facilitates input for users with low literacy or with visual [24], intellectual [25], motor, linguistic, and cognitive disabilities [26] and can support more natural health routine tasks when in-person health care is not possible [19,27]. Third, it opens the door to voice or speech analysis, whereas features of the patient’s utterances can be passively monitored to derive health states [28-31]. Given the lack of agreement on the terminology [6], we will refer to VCAs to indicate the broad technology of dialog apps interacting with humans through speech recognition and synthesis.

VCAs are currently available on 2.5 billion devices worldwide, with smartphones being the leading type of devices, followed by smart speakers and computers. They can be found even in wearable technology, cars, and appliances [32,33]. Moreover, numerous health-related apps of VCAs are available [34]. Thus, these systems are increasingly used in our daily lives and are able to assist in the health care domain. In particular, commercial VCAs such as Amazon Alexa and Google Assistant are increasingly adopted and used as a framework by start-ups and health care organizations to develop products [35-40]. Although there is still room for improvement [41-43], curiosity in using VCAs for health care is growing. VCAs are used to retrieve health-related information (eg, symptoms, medication, nutrition, and health care facilities) [32,44]. This interest is even stronger in low-income households (ie, income <US $50,000 per year). Furthermore, when considering the accessibility of the voice modality for users with low literacy, VCAs could facilitate health management in countries where the education index is still relatively low [45] and smartphones are increasingly penetrating daily life [46] (eg, Brazil, Indonesia, Kenya, Mexico, Philippines, or South Africa).

To the best of our knowledge, only one scoping review has focused on VCAs for health [6]. The authors included research promoting self-management skills and healthy lifestyle behaviors in general and found that, although showing the feasibility of VCAs for health, the evidence was mostly preliminary. However, the authors do not inspect the methodology of the research in enough detail to define the methodological aspects that future research could improve. Thus, our contribution lies in a systematic review of VCA apps dedicated to the prevention and management of chronic and mental health conditions, which aims to provide a broader overview of the current state of research. Thus, we include evidence from both journals and conference papers and provide an overview of aspects affecting technology adoption, that is, system and user performance, ease of use, and attitude toward the target health behavior [47]. Furthermore, we highlight methodological aspects such as variables of interest, instruments used, population tested (in comparison with the target population), and VCA design description.

### Objectives

This study aims to provide a better understanding of the current research on conversational agents delivering health interventions through voice-based interaction and to provide an overview of the methods and evaluations performed. We focus on VCAs specifically dedicated to the prevention and management of chronic and mental health conditions. As we focus on methods and findings in the domain of VCAs, comparing voice modality with others (eg, text and visual) is beyond the scope of this systematic literature review. Therefore, in this study, we seek to answer the following 2 questions: (1) What is the current evidence in favor of VCAs for the prevention and management of chronic and mental health conditions? (2) What are the methods used to evaluate them?

## Methods

### Reporting Standards

This study is compliant with the PRISMA (Preferred Reporting Items for Systematic Reviews and Meta-Analyses) checklist [48] (an overview of the study protocol is given in Multimedia Appendix 1 [49-55]).

### Search Strategy

We conducted a systematic search of the literature available in July 2020 using the electronic databases PubMed MEDLINE, Embase, PsycINFO, Scopus, and Web of Science. These databases were chosen as they cover relevant aspects in the fields of medicine, technology, and interdisciplinary research and have also been used in other systematic reviews covering similar topics [7,8].

Search terms included items describing the constructs *voice modality*, *conversational agent*, and *health* (an overview of the search strategy is given in Multimedia Appendix 2).

### Selection Criteria

We included studies if they (1) were primary research studies involving the prevention, treatment, or management of health conditions related to chronic diseases or mental disorders in patients; (2) involved a conversational agent; (3) the agent used voice as the main interaction modality; and (4) the study included either an empirical evaluation of the system in terms of system accuracy (eg, speech recognition and quality of answers), in terms of technology acceptance (eg, user experience, usability, likability, and engagement), or both.

Papers were excluded if they (1) involved any form of animation or visual representation, for example, embodied agents, virtual humans, or robots; (2) involved any form of health care service via telephone (eg, interactive voice response); (3) focused on testing a machine learning algorithm; and (4) did not target a specific patient population and chronic [49] or mental [50] health conditions.

We also excluded non-English papers, workshop papers, literature reviews, posters, PowerPoint presentations, and papers presented at doctoral colloquia. In addition, we excluded papers of which the authors could not access the full text.

### Selection Process

All references were downloaded and inserted into a Microsoft Excel spreadsheet, and duplicates were removed. A total of 2 independent investigators conducted the screening for inclusion and exclusion criteria in 3 phases: first, we assessed the titles of the records; then their abstracts; and, finally, the full-text papers. After each of these phases, we calculated Cohen kappa to measure the inter-rater agreement between the 2 investigators. The interpretation of the Cohen kappa coefficient was based on the categories developed by Douglas Altman: 0.00-0.20 (poor), 0.21-0.40 (fair), 0.41-0.60 (moderate), 0.61-0.80 (good), and 0.81-1.00 (very good) [56,57]. The 2 raters consulted a third investigator in case of disagreements.

### Data Extraction

A total of 2 investigators extracted data from the eligible papers into a Microsoft Excel spreadsheet with 52 columns containing information on the following aspects: (1) general information about the included papers, (2) voice-based interaction, (3) conversational agents, (4) targeted health conditions, (5) participants, (6) design, (7) measures, (8) main findings, and (9) additional study information such as funding information or conflicts of interest (a complete overview of the study characteristics is given in Multimedia Appendix 3 [52]).

We chose a narrative synthesis of the results and discussed and resolved any inconsistencies in the individual data extractions with a third investigator.

### Risk of Methodological Bias

The choice of an appropriate risk of bias assessment tool was arbitrary, given the prevalence of conference papers and a wide variety of research designs in the included studies. Nevertheless, we wanted to evaluate the selected research concerning the transparency of reporting and the quality of the evidence. After extensive team discussions, the investigators decided to follow the approach of Maher et al [58], who devised a risk of bias assessment tool based on the CONSORT (Consolidated Standards of Reporting Trials) checklist [51]. The tool comprises 25 items and assigns scores of 0 or 1 to each item, indicating if the respective study satisfactorily met the criteria. Higher total scores indicated a lower risk of methodological bias. As the CONSORT checklist was originally developed for controlled trials and no such trials were included in our set of studies, we decided to exclude and adapt certain items as they were considered out of scope for this type of study. We excluded 3.b (*Trial design*), 6.b (*Outcomes*), 7.b (*Sample size*), 12.b (*Statistical methods*), and 14.b (*Recruitment*). Finally, item 17.b (*Outcomes and estimation*) was excluded and 17.a was fragmented into 2 subcriteria (ie, *Provides the estimated effect size* and *Provides precision*). A total of 2 investigators independently conducted the risk of bias assessment, and the differences were resolved in a consensus agreement (details are provided in Multimedia Appendix 4 [51,58]).

## Results

### Selection and Inclusion of Studies

**Figure 1:**
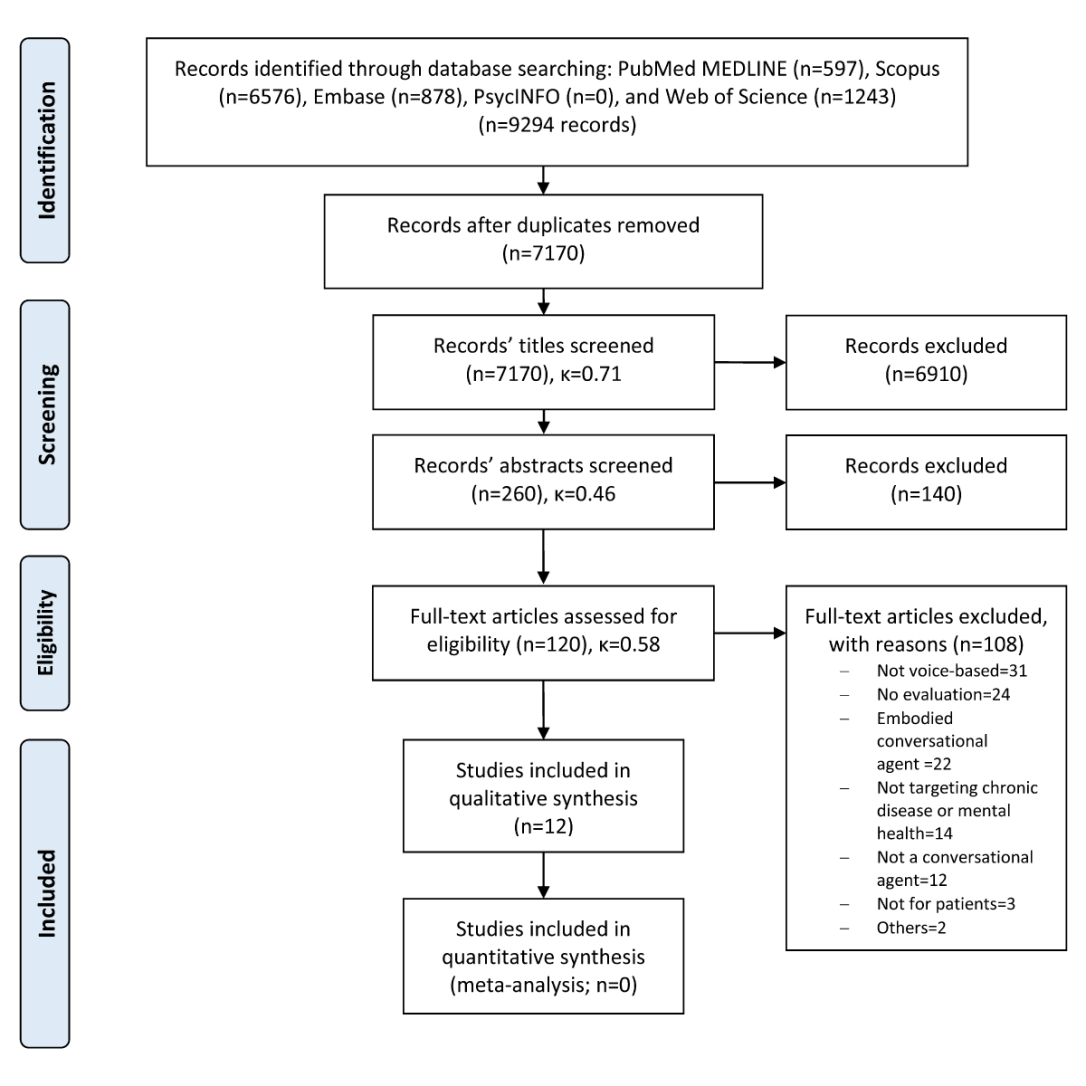
PRISMA (Preferred Reporting Items for Systematic Reviews and Meta-Analyses) flow diagram of included studies.

In total, we screened 7170 deduplicated citations from electronic databases (Figure 1). Of these, we excluded 6910 papers during title screening. We further excluded 140 papers in the abstract screening process, which left us with 120 papers for full-text screening. After assessing the full texts, we found that 108 were not qualified. Cohen kappa was good in titles and full-text screening (κ=0.71 and κ=0.58, respectively), whereas it was moderate in abstract screening (κ=0.46). We explain the latter with a tendency of rater 1 to be more conservative than rater 2, giving a hypothetical probability of chance agreement of 50%. However, after meticulous discussion, the 2 investigators found a balanced agreement (an overview of the reasons for exclusion and the number of excluded records and Cohen kappa are shown in Figure 1) and considered 12 papers as qualified for inclusion and analysis (Table 1).

**Table 1:** Overview and characteristics of included records.

Reference, publication year	Study aim	Type of study participants	Addressed medical condition	Voice-enabled device type	Intervention category
Amith et al (2019) [59]	Development and acceptance evaluation	Healthy adults with at least one child under the age of 18 years (n=16)	Cancers associated with HPV^a^	Tablet	Support
Amith et al (2020) [60]	Development and acceptance evaluation	Healthy young adults aged between 18 and 26 years (n=24)	Cancers associated with HPV	Tablet	Support
Boyd and Wilson (2018) [61]	Criterion-based performance evaluation of commercial conversational agent	Authors as raters (n=2)	Cancers associated with smoking	Smartphone	Support
Cheng et al (2019) [62]	Development and acceptance evaluation	Older adults (n=10)	Diabetes (type 2)	Smart speaker	Monitoring and support
Galescu et al (2009) [63]	Development and performance evaluation	Chronic heart failure patients (n=14)	Heart failure	Not specified	Monitoring
Greuter and Balandin (2019) [64]	Development and performance evaluation	Adults with lifelong intellectual disability (n=9)	Intellectual disability	Smart speaker	Support
Ireland et al (2016) [65]	Development and acceptance evaluation	Adults recruited on campus (n=33)	Parkinson disease, dementia, and autism	Smartphone	Monitoring
Kadariya et al (2019) [66]	Development and acceptance evaluation	Clinicians and researchers (n=16)	Asthma	Smartphone	Monitoring and support
Lobo et al (2017) [67]	Development and acceptance evaluation	Healthy adults working regularly with senior patients (n=11)	Heart failure	Smartphone	Monitoring and support
Ooster et al (2019) [68]	Development and performance evaluation	Normal hearing (n=6)	Hearing impairment	Smart speaker	Monitoring
Rehman et al (2020) [69]	Development and performance and acceptance evaluation	Adults affiliated with the university (n=33)	Diabetes (type 1, type 2, gestational) and glaucoma	Smartphone	Monitoring and support
Reis et al (2018) [70]	Criterion-based performance evaluation of a commercial conversational agent	Not specified (n=Not specified)	Depression	Not specified	Support

^a^HPV: human papillomavirus.

### Characteristics of the Included Studies

The publication years of the selected records ranged between 2009 and 2020, whereas the majority of the papers (n=5) were published in 2019. A total of 7 of the selected records were conference papers and 5 were journal papers.

The majority (n=10) of the selected papers developed and evaluated VCA [59,60,62-69], whereas 2 [61,70] aimed to report a criterion-based performance evaluation of existing commercial conversational agents (eg, Google Assistant and Apple Siri). Among the papers developing and evaluating a VCA, 6 [59,60,62,65-67] assessed the technology acceptance of the VCA, whereas 3 [63,64,68] assessed the system accuracy. Only one [69] assessed both performance and acceptance.

All studies (n=12) were nonexperimental [59-70], that is, they did not include any experimental manipulation. A total of 4 papers [61,66,68,70] did not explicitly specify the study design they used, whereas the other papers provided labels. One study stated conducting a *feasibility evaluation* [63], 1 a *focus group study* [65], 1 a *qualitative assessment of effectiveness and satisfaction* [62], and 1 a *case study* [69]. Furthermore, 1 conducted a *pilot study* [64], 2 declared deploying a *Wizard-of-Oz* (WOz) experiment [59,60], and 1 a *usability study* [67].

An overview of the included studies can be found in Table 1 (all details in Multimedia Appendix 3).

### Main Findings

#### System Accuracy

Half (n=6) of the included studies [61,63,64,68-70] evaluated the accuracy of the system. In total, 4 of those studies [63,64,68,69] described precise speech recognition performance, whereas 3 [63,68,69] reported good or very good speech recognition performance, and 1 [64] study found mediocre recognition accuracy, with single-letter responses being slightly better recognized than word-based responses (details on speech recognition performance are given in Multimedia Appendix 5 [52]). A total of 2 studies [61,70] qualitatively assessed the accuracy of the VCAs. One study [61] observed that the standard Google Search performs better than a voice-activated internet search performed with Google Assistant and Apple Siri. Another study [70] reported on the accuracy of assisting with social activities. They observed all commercial VCAs to perform well at basic greeting activities, Apple Siri and Amazon Alexa to perform the best in email management, and Apple Siri to perform the worst in supporting social games. Moreover, Google Assistant performed the best in social game activities but the worst in social media management.

#### Technology Acceptance

Of the 12 studies, 7 [59,60,62,65-67,69] reported technology acceptance findings, whereas the others (n=5) did not [61,63,64,68,70]. A total of 3 studies [60,66,67] reported technology acceptance through a System Usability Survey (SUS). One study [67] reported a relatively high usability score (SUS score of mean 88/100), whereas 1 study [60] described better usability of its VCA for human papillomavirus (HPV) in comparison with industry standards (ie, SUS score of mean 72/100). The latter also compared SUS scores between groups and found a higher score for participants who did not receive the HPV vaccine (mean 80/100), compared with those who did (mean 77/100) and the control group (mean 74/100). Note that the SDs of these results were not provided. In addition, the study found the score of Speech User Interface Service Quality to be medium (mean 4.29/7, SD 0.75). The third study [66] asked clinicians and researchers to evaluate the VCA with broader set of results. Clinicians and researchers rated the VCA with very good usability (ie, SUS score of mean 83.13/100 and 82.81/100, respectively) and very good naturalness (mean 8.25/10 and 8.63/10, respectively), information delivery (mean 8.56/10 and 8.44/10, respectively), interpretability (mean 8.25/10 and 8.69/10, respectively), and technology acceptance (mean 8.54/10 and 8.63/10, respectively). SDs of these results were not reported. A total of 2 studies [59,69] have reported different types of evaluations of technology acceptance. Thus, 1 study [59] reported good ease of use (mean 5.4/7, SD 1.59) and acceptable expected capabilities (mean 4.5/7, SD 1.46) but low efficiency (mean 3.3/7, SD 1.85) of its VCA, whereas the other [69] described a positive user experience of its VCA with all User Experience Questionnaire constructs. As the authors provided User Experience Questionnaire mean values per item we could only infer the mean values per construct manually. That is, Attractiveness mean score was 1.88/3; Perspicuity mean score was 1.93/3; Efficiency mean score was 1.88/3; Dependability mean score was 1.70/3; Stimulation mean score was 1.90/3; and Novelty mean score was 1.85/3. Note that the SDs of these results were not provided. Finally, 2 studies reported a qualitative evaluation of their VCA, one [62] stating theirs to be *more accepted than rejected* in terms of user satisfaction, without giving more details, and the other [65] mentioning a general *positive impression* but a slowness in the processing of their VCA.

### Methodology of the Included Studies

We included all types of measures that were present in more than 1 study, that is, system accuracy measures, technology acceptance measures, behavioral measures, measures of attitude toward the target health behavior, and reported previous experience with technology.

The majority of the studies (n=10) did not report any behavioral measures [59-63,65-67,69,70], whereas 2 papers [64,68] did. One [68] described the frequency of verbal responses not relevant to the system (ie, nonmatrix-vocabulary words), whereas the other [64] provided engagement and user performance (ie, task completion, time to respond, points of difficulty, points of dropout, and quality of responses).

Half of the studies (n=6) did not report on any system measures [59,60,62,65-67], whereas the other half reported either speech recognition performance measures (n=4) [63,64,68,69] or criterion-based evaluation of the goodness of the VCA’s response (n=2) [61,70]. In particular, 4 studies [63,64,68,69] measured speech recognition performance compared with human recognition. One of those [68] measured the accuracy of a diagnostic test score (ie, speech reception threshold) compared with the manually transcribed results. One study [64] measured the speech recognition percentage inferred from transcriptions of the interaction. One study [63] compared the VCA with nurse practitioners’ interpretations of patients’ responses. Finally, 1 [69] study gave more detailed results, reporting a confusion matrix; speech recognition accuracy, precision, sensitivity, specificity, and F-measure; and performance in task completion rate and prevention from security breaches.

Of the 12 studies, 7 [59,60,62,65-67,69] reported technology acceptance measures, whereas the remaining studies [61,63,64,68,70] did not. Although 2 studies [60,69] used validated questionnaires only and 2 [62,67] used adapted questionnaires only, 1 study used both validated and adapted questionnaires [66]. One study [59] used an adapted questionnaire and qualitative feedback as acceptance measures. One study [65] reported only qualitative feedback.

The majority of the included studies (n=10) did not provide measures of attitude toward the target health behavior [61-70]. The 2 remaining papers [59,60] provided validated questionnaires, and both focused on attitudes toward HPV vaccines. One study [59] used the Parent Attitudes about Childhood Vaccines, and 1 study [60] used the Carolina HPV Immunization Attitude and Belief Scale.

The majority of the included studies (n=8) also did not report controlling for participants’ previous experience with technology [59-63,66,69,70]. Of the remaining 4 studies, 1 study [68] reported that all study participants had no experience with smart speakers; 1 [67] reported that all study participants were familiar with mobile health apps; and 1 [65] controlled for participants’ smartphone ownership, use competence on Androids, iPhones, tablets, laptops, and desktop computers. Finally, 1 study [64] assessed the previous exposure of study participants to voice-based assistants but did not report on the results.

In general, risk bias varied markedly, from a minimum of 1 [70] to a maximum of 11.25 [60] and a median of 6.36 (more details are provided in Multimedia Appendix 4).

### Health Characteristics

Of the included studies, cancer was the most common health condition targeted; 2 papers [59,60] addressed cancer associated with HPV, whereas 1 study [61] addressed cancer associated with smoking. The next most commonly addressed conditions were diabetes (n=2) [62,69] and heart failure (n=2) [63,67]. Other discussed conditions were hearing impairment [68], asthma [66], and Parkinson disease [65]. A total of 3 papers addressed psychological conditions [64,65,70]. Specifically, they focused on dementia and autism [65], intellectual disability [64], and depression [70].

When inspecting the target population, we observed that 3 of the included studies [62,67,70] targeted older people, whereas 2 targeted either parents of adolescents [59] or pediatric patients [60]. The others targeted hearing-impaired individuals [68], smokers [61], patients with asthma [66], patients with glaucoma and diabetes [69], people with intellectual disability [64], and patients with chronic heart failure [63]. One study [65] did not specify a particular target population.

The actual study participants consisted of the following samples: healthy adults with at least one child under the age of 18 years (N=16) [59], healthy young adults aged between 18 and 26 years (N=24) [60], the authors themselves (N=2) [61], older adults (N=10) [62], patients with chronic heart failure (N=14) [63], adults with lifelong intellectual disability (N=9) [64], adults recruited on campus (N=33) [65], clinicians and researchers (N=16) [66], healthy adults working regularly with senior patients (N=11) [67], normal-hearing people (N=6) [68], and adults affiliated with a university (N=33) [69]. One study [70] did not specify the type or number of participants.

### Characteristics of VCAs

A total of 8 studies [60,62,63,65-69] named their VCA, whereas 2 studies [59,64] did not specify any name (Multimedia Appendix 5). In total, 2 studies [61,70] did not provide a name because they evaluated existing commercially available VCA (ie, Amazon Alexa, Microsoft Cortana, Google Assistant, and Apple Siri).

The majority of the included studies (n=7) did not describe the user interface of their VCAs [60-62,64,68,70], whereas the remaining 5 papers did [59,65-67,69].

The underlying architecture of the investigated VCAs was described in 7 of the included studies [62,63,66-70], whereas 3 papers did not provide this information [61,64,65]. A total of 2 studies [59,60] could not provide any architectural information, given the nature of their study design (ie, WOz).

When considering the devices used to test the VCA, we found that smartphones were the most used (n=5) [61,65-67,69], followed by smart speakers (n=3) [62,64,68] and tablets (n=2) [59,60]. A total of 2 studies [63,70] did not specify which device they used for data collection.

The vast majority of the VCAs (n=10) were not commercially available [59,60,62-69] at the time of this systematic literature review. In particular, 1 study [65] reported the VCA to be available on Google Play store at the time of publication; however, the app could not be found by the authors of this literature review at the time of reporting (we controlled for geo-blocking by searching the app with an internet protocol address of the authors’ country of affiliation [65]). Given that the other 2 studies tested consumer VCA, we classified these papers as testing commercially available VCAs [61,70].

### Characteristics of Voice-Based Interventions

Interventions were categorized as either monitoring, support or both. Monitoring interventions refer to those focusing on health tracking (eg, symptoms and medication adherence), whereas support interventions include targeted or on-demand information or alerts. This categorization was based on the classification of digital health interventions by the World Health Organization [52]. A total of 5 VCAs [59-61,64,70] exclusively focused on support, and 3 studies [63,65,68] exclusively focused on monitoring. In total, 4 studies investigated a VCA providing both monitoring and support [62,66,67,69]. Monitoring activities were mainly implemented as active data capture and documentation (n=5) [62,63,66-69], whereas 1 study [66] also focused on self-monitoring of health or diagnostic data. One study [65] investigated self-monitoring of health or diagnostic data as the main monitoring activity.

Support services mainly consisted of delivering targeted health information based on health status (n=4) [59,60,64,67,69], whereas 1 study [67] also provided a lookup of health information. A total of 3 studies provided such a lookup of health information only [61,62,66], whereas 2 [62,66] also provided targeted alerts and reminders. Finally, 1 study delivered a support intervention in the form of task completion assistance [70] (more details on the interventions are given in Multimedia Appendix 3).

## Discussion

### Principal Findings

The goal of this study is to summarize the available research on VCA for the prevention and management of chronic and mental health conditions and provide an overview of the methodology used. Our investigation included 12 papers reporting studies on the development and evaluation of a VCA in terms of system accuracy and technology acceptance. System accuracy refers to the ability of the VCA to interact with the participants, either in terms of speech recognition performance or in terms of the ability to respond adequately to user queries. Technology acceptance refers to all measures of the user’s perception of the system (eg, user experience, ease of use, and efficiency of interaction).

Most of the studies reported either one or the other aspect, whereas only 1 study reported both aspects. In particular, speech recognition in VCA prototypes was mostly good or very good. The only relevant flaw revealed was a slowness in the VCA responses, reported in 2 of the selected studies [59,65]. Commercial VCAs, although not outperforming Google Search when the intervention involved lookup of health information, seem to have a specialization in supporting certain social activities (eg, Apple Siri and Amazon Alexa for social media and office-related activities and Google Assistant for social games). These results suggest that there is great potential for noncommercial VCAs, as they perform well in the domain for which they were built, whereas commercial VCAs are rather superficial in their health-related support. Moreover, despite the heterogeneity of technology acceptance measures, the results showed good to very good performance. This suggests that the reviewed VCAs could satisfy users’ expectations when supporting the prevention and management of chronic or mental conditions. The evidence remains, however, hard to be conclusive. In fact, the majority of the included studies were published relatively recently, around 2019, and were fairly distributed between journal and conference or congress papers. Moreover, all studies were nonexperimental, and there was a general heterogeneity in the evaluation methods, especially in the user perception of the technology (ie, user experience). In particular, only 3 [60,66,69] of the 7 studies that included a measure of technology acceptance through a questionnaire [59,60,62,65-67,69] used a validated questionnaire, whereas the others adapted them. There was also a general discrepancy between the target population and the actual sample recruited. In particular, although the VCAs studied were dedicated to the management or prevention of chronic and mental health conditions, the evaluation was mainly conducted with healthy or convenience samples. Finally, according to our risk of bias assessment, the evidence is generally reported with insufficient transparency, leaving room for doubt about the generalizability of results, both in terms of technical accuracy and technology acceptance.

Considering the aforementioned aspects and the limited number of studies identified, it seems that research on VCAs for chronic diseases and mental health conditions is still in its infancy. Nevertheless, the results of almost all studies reporting system accuracy and technology acceptance are encouraging, especially for the developed VCAs, which inspires further development of this technology for the prevention and management of chronic and mental health conditions.

### Related Work

To the best of our knowledge, this is the only systematic literature review addressing VCAs specifically dedicated to the prevention and management of chronic diseases and mental illnesses. Only 1 scoping review appraised existing evidence on voice assistants for health and focused on interventions of healthy lifestyle behaviors in general [6]. The authors highlight the importance of preventing and managing chronic diseases; however, although they report the preliminary state of evidence, they do not stress, for instance, specific methodological aspects that future research should focus on, to provide more conclusive evidence (eg, test on the actual target population). Moreover, the authors did not provide a measure of the preliminary state of evidence. However, it is important to inspect what aspects of the studies are most at risk of bias, to allow for a clearer interpretation of the results. Our review aims to highlight these aspects to provide meaningful evidence, not only for the scientific community in the field of disease prevention but also for this broad study population. We aimed to identify as precisely as possible the methodological gaps, to provide a solid base upon which future research can be crafted upon. For this reason, we first provide an overview of the instruments used and the variables of interest, distinguishing between behavioral and system and technology acceptance measures (compared with the sole outcome categorization), providing a more fine-grained overview of the methods used. Second, we provide a stronger argument in favor of the potential bias present in the research and, thus, the difficulty in interpreting the existing evidence, with a critical appraisal of the methodology, through a risk bias assessment. Moreover, the authors [6] included studies investigating the technology acceptance but excluded studies providing evidence on the technical performance of VCAs. However, this aspect has an important influence on the technology acceptance [71]. Thus, our review highlights the current state of research not only on the user’s perception (ie, technology acceptance) but also on the device’s ability to interact with the user (ie, technical performance). These aspects allowed us to provide a fair profile of the studies and to draw stronger conclusions on the methodology used to study a group of VCAs promoting the prevention and management of chronic diseases and mental illnesses.

Our findings are coherent with the review by Sezgin et al [6] in a series of aspects. First, we also show that research on VCAs is still emerging, with studies including small samples and focusing on the feasibility of dedicating VCA for a specific health domain. Second, we also find a heterogeneous set of target populations and target health domains. However, our findings are in contrast with those of Sezgin et al [6] in the following aspects. First, we report studies mainly focusing on developing and evaluating the system in terms of system accuracy or technology acceptance; Sezgin et al [6] also described efficacy tests but did not report on system accuracy. Third, the papers included in this study presented only VCA apps, whereas Sezgin et al [6] also included automated interventions via telephone. Finally, despite the preliminary character of the research, we include a risk bias assessment to formalize the importance of rigorous future research on VCAs for health.

In general, as we tried to include results explaining the technology acceptance of VCAs as a digital health intervention for the prevention and management of chronic and mental health conditions, our findings are more appropriate when concluding the current evidence-based VCAs in this specific domain rather than in healthy lifestyle behaviors in general.

### Limitations

There are several limitations to our study, which may limit the generalizability of our results. First, our search strategy focused on nonspecific constructs (eg, health), which may have led to the initial inclusion of a large number of unrelated literature, in addition to that concerning the main topic of this review (ie, VCAs for chronic diseases and mental health). Given the infancy of this field, however, we chose a more inclusive strategy to avoid missing relevant literature for the analysis. Second, our systematic literature review aimed to assess the current scientific evidence in favor of VCAs for chronic diseases and mental health, thus not encompassing the developments of this technology in the industry. However, we aimed to summarize the findings and current methodologies used in the research domain and provided an overview of the scientific evidence on this technology. Third, to evaluate a possible experimental bias of the studies, we followed the reporting guidelines suggested by the *Journal of Medical Internet Research* and chose the CONSORT-EHEALTH checklist. Risk bias varied significantly among the selected studies. This evaluation scheme may be regarded as unsuitable for evaluating the presented literature, as none of the papers reported an experimental trial. An evaluation scheme capable of taking into account the pioneering character of the papers concerning the use of this technology for health-related apps could have enabled a more differentiated assessment.

### Future Work

The wide adoption of voice assistants worldwide and the interest in using them for health care purposes [32] have generated great potential for the effective implementation of scalable digital health interventions. There is, however, a lack of a clear implementation framework for VCAs. For instance, text-based and embodied conversational agents can currently be implemented using existing frameworks dedicated to digital health interventions [72-75]; however, to the best of our knowledge, there is no such framework for VCAs. A platform for the development of VCAs dedicated to specific chronic or mental health conditions could encourage standardized implementation, which would be more comparable in their development and evaluation processes. Currently, it is possible to develop apps for consumer voice assistants (eg, *skills* for Amazon Alexa or *actions* for Google Assistant). However, these products may be of privacy [76] or safety concerns [77]. Therefore, the academic community should strive for the creation of such a platform to foster the development of VCA for health.

The identified research provides diverse and general evaluation measures around technology acceptance (or user experience in general) and no evaluation based on theoretical models of health behavior (eg, intention of use). Thus, although the developed VCA might have been well received by the studied population samples, there is a need for a more systematic and comparable evaluation of the evidence systems to understand which aspects of VCAs are best for user satisfaction. Future research should favor the use of multiple standardized questionnaires dedicated to voice user interfaces [78] to further explore the factors potentially influencing their effectiveness (eg, rapport [79] and intention of use [71]).

This study reported the current state of research in the specific domain of VCAs for the prevention and management of chronic and mental health conditions in terms of behavioral, technological accuracy, and technology acceptance measures. However, the question remains as to how voice modality performs on these variables in comparison with other modalities, such as text-based conversational agents. Text-based conversational agents have been extensively studied in the domain of digital health interventions [80-83] and can be considered as a precursor to VCAs [9]. Moreover, voice modality may differ in their appropriateness of app, compared with text modality, depending on the health-related context (eg, public spaces [84,85] and type of user [24-26,86,87]). Thus, future research should not only standardize the research in terms of implementation and evaluation measures but also consistently evaluate this technology against what we could consider the gold standard of conversational agents.

Moreover, only 4 papers [63,64,68,69] compared the accuracy of the VCA’s interpretation of participants’ responses with humans’ interpretation of participants’ responses. Although it was limited to speech recognition, they were the only cases of human-machine comparison. To verify the suitability of VCAs as an effective and scalable complementary alternative to health care practitioners, more research should compare not only the system accuracy but also the general performance of this type of digital health intervention in comparison with standard in-person health care.

Finally, all papers conducted laboratory experiments and focused on short-term performance and technology acceptance. Even if this evidence shows the feasibility of VCAs for health care, it does not provide evidence on the actual effectiveness of VCAs in assisting patients in managing their chronic and mental health conditions compared with standard practices. Future research should provide evidence on complementary short-term and long-term measurements of technology acceptance and behavioral and health outcomes associated with the use of VCAs.

### Conclusions

This study provides a systematic review of VCAs for the prevention and management of chronic and mental health conditions. Out of 7170 prescreened papers, we included and analyzed 12 papers reporting studies either on the development and evaluation of a VCA or on the criterion-based evaluation of commercial VCAs. We found that all studies were nonexperimental, and there was general heterogeneity in the evaluation methods. Considering the recent publication date of the included papers, we conclude that this field is still in its infancy. However, the results of almost all studies on the performance of the system and the experiences of users are encouraging. Even if the evidence provided in this study shows the feasibility of VCAs for health care, this research does not provide any insight into the actual effectiveness of VCAs in assisting patients in managing their chronic and mental health conditions. Future research should, therefore, especially focus on the investigation of health and behavioral outcomes, together with relevant technology acceptance outcomes associated with the use of VCAs. We hope to stimulate further research in this domain and to encourage the use of more standardized scientific methods to establish the appropriateness of VCAs in the prevention and management of chronic and mental health conditions.

## Appendix

Multimedia Appendix 1 Study protocol.

Multimedia Appendix 2 Search terms per construct (syntax used in PubMed Medline).

Multimedia Appendix 3 Complete list of characteristics of the included studies.

Multimedia Appendix 4 Risk-of-bias assessment.

Multimedia Appendix 5 Main characteristics of the included studies.

## Abbreviations

CONSORT: Consolidated Standards of Reporting Trials
HPV: human papillomavirus
SUS: System Usability Survey
VCA: voice-based conversational agent
WOz: Wizard-of-Oz
