# Supplementary material for: Voice-Based Conversational Agents for the Prevention and Management of Chronic and Mental Health Conditions: Systematic Literature Review
Source: J Med Internet Res. 2021 Mar 29;23(3):e25933. doi: 10.2196/25933 (PMC8042539; doi:10.2196/25933)
Supplement: Multimedia Appendix 1 [file jmir_v23i3e25933_app1.pdf]

Multimedia Appendix: Study protocol

Bérubé et al., 2021

Adopted from PRISMA-P (Preferred Reporting Items for Systematic review and Meta-Analysis Protocols) and PROSPERO.

This is a Multimedia Appendix to a full manuscript published in the J Med Internet Res. For full copyright and citation information see http://dx.doi.org/10.2196/jmir.25933.

**Topic**	**Content**
Title	Voice-Based Conversational Agents for the Prevention and Management of Chronic and Mental Health Conditions: Systematic Literature Review
Authors	Caterina Bérubé, Theresa Schachner, Roman Keller, Elgar Fleisch, Florian von Wangenheim, Filipe Barata, Tobias Kowatsch
Review team members and their organizational affiliations	• Caterina Bérubé:<br>1. Centre for Digital Health Interventions, Department of Management, Technology, and Economics, ETH Zurich, Zurich, Switzerland<br>• Theresa Schachner:<br>1. Centre for Digital Health Interventions, Department of Management, Technology, and Economics, ETH Zurich, Zurich, Switzerland<br>• Roman Keller:<br>1. Future Health Technologies Programme, Campus for Research Excellence and Technological Enterprise (CREATE), Singapore-ETH Centre, Singapore, Singapore<br>• Prof. Dr. Elgar Fleisch:<br>1. Centre for Digital Health Interventions, Department of Management, Technology, and Economics, ETH Zurich, Zurich, Switzerland;<br>2. Future Health Technologies Programme, Campus for Research Excellence and Technological Enterprise (CREATE), Singapore-ETH Centre, Singapore, Singapore;<br>3. Centre for Digital Health Interventions, Institute of Technology Management, University of St. Gallen, St. Gallen, Switzerland<br>• Prof. Dr. Florian von Wangenheim:<br>1. Centre for Digital Health Interventions, Department of Management, Technology, and Economics, ETH Zurich, Zurich, Switzerland;<br>2. Future Health Technologies Programme, Campus for Research Excellence and Technological Enterprise (CREATE), Singapore-ETH Centre, Singapore, Singapore<br>• Dr. Filipe Barata:<br>1. Centre for Digital Health Interventions, Department of Management, Technology, and Economics, ETH Zurich, Zurich, Switzerland<br>• Dr. Tobias Kowatsch:<br>1. Centre for Digital Health Interventions, Department of Management, Technology, and Economics, ETH Zurich, Zurich, Switzerland;

	2. Future Health Technologies Programme, Campus for Research Excellence and Technological Enterprise (CREATE), Singapore-ETH Centre, Singapore, Singapore;<br>3. Centre for Digital Health Interventions, Institute of Technology Management, University of St. Gallen, St. Gallen, Switzerland;<br>4. Saw Swee Hock School of Public Health, National University of Singapore, Singapore, Singapore
Contact details for of corresponding author	Caterina Bérubé, MSc<br>Centre for Digital Health Interventions<br>Department of Management, Technology, and Economics<br>ETH Zurich<br>WEV G 214<br>Weinbergstrasse 56/58<br>Zurich, 8092<br>Switzerland<br>Phone: +41 44 633 8419<br>Email: cberube@ethz.ch
Organizational affiliation of the review	Centre for Digital Health Interventions, https://www.c4dhi.org/
Type and method of review	Systematic review
Contributions	CB, EF, and TK were responsible for the study design and search strategy. CB and RK were responsible for the screening and data extraction. CB, RK, and TS were responsible for the data analysis. CB, RK, TS, and FB were responsible for the first draft. All authors were responsible for critical feedback and final revisions of the manuscript. TS and RK share second authorship. FB and TK share last authorship.
Sources/Sponsors	This work was supported by the National Research Foundation, Prime Minister's Office, Singapore, under its Campus for Research Excellence and Technological Enterprise Programme and by the CSS Insurance (Switzerland).
Conflict of interest	All authors are affiliated with the Centre for Digital Health Interventions (www.c4dhi.org), a joint initiative of the Department of Management, Technology, and Economics at ETH Zurich and the Institute of Technology Management at the University of St. Gallen, which is funded in part by the Swiss health insurer CSS. EF and TK are also the cofounders of Pathmate Technologies, a university spin-off company that creates and delivers digital clinical pathways. However, Pathmate Technologies was not involved in any way in the design, interpretation, analysis, or writing of the study.
Rationale	In this systematic literature review, we seek to answer the following 2 questions:<br>(1) What is the current evidence in favor of VCAs for the prevention and management of chronic and mental health conditions?<br>(2) What are the methods used to evaluate them?
Eligibility criteria	Inclusion criteria:<br>(1) Primary research studies that involved the prevention, treatment, or management of health conditions related to chronic diseases or mental disorders in patients<br>(2) Involvement of a conversational agent<br>(3) The agent was based on voice as the main interaction modality

	(4) Inclusion of an empirical evaluation of the system<br><br>Exclusion criteria:<br><br>(1) Involvement of any form of animation or visual representation, e.g. embodied agents, virtual humans, or robots<br>(2) Involvement of any form of healthcare service via telephone<br>(3) Focus on testing a machine learning algorithm<br>(4) Not targeting a specific patient population, chronic disease (defined by the WHO categories) [1] or mental health condition (defined by the WHO) [2].
Information sources	A systematic database search will be conducted accessing PubMed MEDLINE, Embase, PsycINFO, Scopus, and Web of Science. The databases were chosen as they cover relevant topics in the fields of medicine (PubMed MEDLINE, Embase), technology and interdisciplinary research (PsycINFO, Scopus, and Web of Science). The search terms include synonyms, acronyms and commonly known terms of the constructs: “Voice modality”, “Conversational agent” and “Healthcare”. Grey literature such as presentations, posters, dissertations and theses will be excluded.
Search strategy	Search strategy for PubMed Medline<br>(https://pubmed.ncbi.nlm.nih.gov/)<br>Filters: none<br>Conducted in July 2020<br>("voice" OR "voice-based" OR "voice control*" OR "voice command*" OR "voice prompt*" OR "voice-activated" OR "speech" OR "speech recognition" OR "voice-assisted" OR "conversational" OR "dialogue" OR "text-to-speech") AND ("agent" OR "assistant*" OR "intelligent personal assistant*" OR "virtual personal assistant*" OR "virtual assistant*" OR "automated personal assistant*" OR "smart-home control" OR "smart speaker*" OR "digital assistant*" OR "user interface*" OR "interface*" OR "google assistant" OR "alexa" OR "siri" OR "chatbot" OR "personal digital assistant" OR "dedicated personal assistant" OR "virtual assistant") AND ("health intervention" OR "intervention" OR "health" OR "healthcare" OR "digital health" OR "ubiquitous health" OR "uhealth" OR "mhealth" OR “health behavio?r” OR "emergency" OR "mental health")
Type of included study	Any type of primary research
Studied domain	Digital health with a focus on chronic conditions and mental health
Population/Participants	Clinical (chronic or mental health conditions) population, and non-clinical population and participants
Data collection and selection process	Two independent reviewers will conduct the initial screening of the searched studies based on their titles and abstracts. The same reviewers will then independently conduct the full-text screening based on the eligibility/inclusion criteria. Cohen kappa will be computed after each step (title screening, abstract screening, full-text screening) to measure interrater agreement. Any disagreement will be discussed in person. If no consensus can be reached, a third reviewer will be consulted to achieve an agreement.
Data items for coding	The following data items will be extracted of each included study: paper information (authors, title, permalink, year of publication, paper type, journal/outlet), study aim, intervention (category, sub-category [3],

	description, features included), voice-based conversational agent (VCA; whether the user interface is described, whether the architecture is described, name, software implementation, hardware implementation device and device model, commercial availability), health-related information (target illness, target population), design (type, sub-type), participants (sample size, recruitment pool, demographics, age, health status, previous experience with technology, previous experience with VCAs), measures (technology acceptance, constructs or sub-categories of technology acceptance, system accuracy, behavior, attitude towards the target health behavior), findings (technology acceptance in summary and in details, system accuracy in summary and in details, behavior in summary and in details), additional information (discussed limitations, funding source, conflict of interest, study location, study continent). Non-available data will be marked with a minus sign.
Outcomes and prioritization	Main outcomes: Any voice-based-related and conversational-agent-related intervention outcomes (e.g. aim of the voice-based intervention, characteristics of the voice-based intervention, application implementation), any healthcare-related intervention outcomes (e.g. target illness, target complication, target population)<br>Additional outcomes: Any evaluation related outcomes (e.g. attitude towards the system, attitude towards health behavior, interaction behavior, system performance)
Risk of bias in individual studies	Two independent researchers will assess the risk of bias of the included studies. Possible candidate tools for analysis will be the Cochrane risk of bias tool or the application of risk of bias assessment checklists based on the CONSORT checklist [4]. Additionally, the application of a tool to assess the quality of the included studies will be discussed by the study authors and applied if appropriate.
Data synthesis	The PRISMA statement will be followed for data synthesis and a narrative synthesis of the included studies will be performed.
Language	English
Country	Switzerland
Anticipated or actual start date	July 2020
Anticipated or actual end date	November 2020

Adopted from:

Booth A, Clarke M, Dooley G, Ghersi D, Moher D, Petticrew M, Stewart L. The nuts and bolts of PROSPERO: aninternational prospective register of systematic reviews. Syst Rev 2012;1(1):2. PMID:22587842

Moher D, Shamseer L, Clarke M, Ghersi D, Liberati A, Petticrew M, Shekelle P, Stewart LA, PRISMA-P Group. Preferred reporting items for systematic review and meta-analysis protocols (PRISMA-P) 2015 statement. Syst Rev 2015;4(1):1. PMID:25554246

Shamseer L, Moher D, Clarke M, Ghersi D, Liberati A, Petticrew M, Shekelle P, Stewart LA, PRISMA-P Group. Preferred reporting items for systematic review and meta-analysis protocols (PRISMA-P) 2015: elaboration and explanation. BMJ 2015;350(jan02 1):g7647. PMID:25555855

References

1. WHO | Fact sheets: chronic diseases. Published online 2014. Accessed November 22, 2020. https://www.who.int/topics/chronic_diseases/factsheets/en/

2. WHO | Fact sheets: mental disorders. Who.int. Published online 2019. Accessed November 22, 2020. https://www.who.int/news-room/fact-sheets/detail/mental-disorders

3. WHO. Classification of digital health interventions v1.0: A shared language to describe the uses of digital technology for health. 2018 WHO/RHR/19.06. https://apps.who.int/iris/bitstream/handle/10665/260480/WHO-RHR-18.06-eng.pdf?sequence=1

4. Schulz KF, Altman DG, Consort MD. CONSORT 2010 statement: updated guidelines for reporting parallel group randomised trials. BMJ. 2010 2010;2010;340:c332. PMID: 20352064.
