# Supplementary material for: Voice-Based Conversational Agents for the Prevention and Management of Chronic and Mental Health Conditions: Systematic Literature Review
Source: J Med Internet Res. 2021 Mar 29;23(3):e25933. doi: 10.2196/25933 (PMC8042539; doi:10.2196/25933)
Supplement: Multimedia Appendix 2 [file jmir_v23i3e25933_app2.pdf]

Multimedia Appendix: Search terms per construct (syntax used in PubMed Medline)

This is a Multimedia Appendix to a full manuscript published in the J Med Internet Res. For full copyright and citation information see http://dx.doi.org/10.2196/jmir.25933.

Search category	Search terms
**1. Voice modality**	“voice" OR "voice-based" OR "voice control*" OR "voice command*" OR "voice prompt*" OR "voice-activated" OR "speech" OR "speech recognition" OR "voice-assisted" OR "conversational" OR "dialogue" OR "text-to-speech"
**2. Conversational agent**	"agent" OR "assistant*" OR "intelligent personal assistant*" OR "virtual personal assistant*" OR "virtual assistant*" OR "automated personal assistant*" OR "smart-home control" OR "smart speaker*" OR "digital assistant*" OR "user interface*" OR "interface*" OR "google assistant" OR "alexa" OR "siri" OR "chatbot" OR "personal digital assistant" OR "dedicated personal assistant" OR "virtual assistant"
**3. Health**	"health intervention" OR "intervention" OR "health" OR "healthcare" OR "digital health" OR "ubiquitous health" OR "uhealth" OR "mhealth" OR “health behavio?r” OR "emergency" OR "mental health"
**4. Combined**	1 AND 2 AND 3
