# Supplementary material for: Voice-Based Conversational Agents for the Prevention and Management of Chronic and Mental Health Conditions: Systematic Literature Review
Source: J Med Internet Res. 2021 Mar 29;23(3):e25933. doi: 10.2196/25933 (PMC8042539; doi:10.2196/25933)
Supplement: Multimedia Appendix 3 [file jmir_v23i3e25933_app3.pdf]

Multimedia Appendix: Complete list of characteristics of the included studies

This is a Multimedia Appendix to a full manuscript published in the J Med Internet Res. For full copyright and citation information see http://dx.doi.org/10.2196/jmir.25933.

Paper				
Authors	Title	Year of publication	Type	Outlet
M Amith, A. Zhu, R. Cunningham, R. Lin, L. Savas, L. Shay, Y. Chen, Y. Gong, J. Boom, K. Roberts, C. Tao	Early Usability Assessment of a Conversational Agent for HPV Vaccination	2019	Journal paper	Studies in Health Technology and Informatics
M. Amith, R. Lin, R. Cunningham, Q. Wu, L. Savas, Y. Gong, J. Boom, L. Tang, C. Tao	Examining Potential Usability and Health Beliefs Among Young Adults Using a Conversational Agent for HPV Vaccine Counseling	2020	Congress paper	AMIA Joint Summits on Translational Science proceedings
M. Boyd, N. Wilson	Just ask Siri? A pilot study comparing smartphone digital assistants and laptop Google searches for smoking cessation advice	2018	Journal paper	PloS one
A. Cheng, V. Raghavaraju, J. Kanugo, Y.P. Handrianto, Y. Shang	Development and Evaluation of a Healthy Coping Voice Interface Application Using the Google Home for Elderly Patients with Type 2 Diabetes	2019	Conference paper	2018 15th IEEE Annual Consumer Communications & Networking Conference
L. Galescu, J. Allen, G. Ferguson, J. Quinn, M. Swift	Speech recognition in a dialog system for patient health monitoring	2009	Conference paper	IEEE International Conference on Bioinformatics and Biomedicine (BIBM09)
S. Greuter, S. Balandin	Social Games Are Fun: Exploring Social Interactions on Smart Speaker Platforms for People with Disabilities	2019	Conference paper	CHI PLAY EA '19
D. Ireland, C. Atay, J. Liddle, D. Bradford, H. Lee, O. Rushin, T. Mullins, D. Angus, J. Wiles, S. McBride, A. Vogel	Hello harlie: Enabling speech monitoring through chat-bot conversations	2016	Journal paper	Studies in health technology and informatics
D. Kadariya, R. Venkataramanan, M. Kalra, K. Thirunarayan	kBot: Knowledge-Enabled Personalized Chatbot for Asthma Self-Management	2019	Conference paper	2019 IEEE International Conference on Smart Computing (SMARTCOMP)
J. Lobo, L. Ferreira, A.J. Ferreira	CARMIE: A conversational medication assistant for heart failure	2017	Journal paper	International Journal of E-Health and Medical Communications
J.Ooster, P.N. Porysek Moreta, J.-H. Bach, I. Holube, B.T. Meyer	"Computer, test my hearing": Accurate speech audiometry with smart speakers	2019	Conference paper	INTERSPEECH 2019
U.U. Rehman, D.J. Chang, Y. Jung, U. Akhtar, M.A. Razzaq, S. Lee	Medical Instructed Real-Time Assistant for Patient with Glaucoma and Diabetic Conditions	2020	Journal paper	Applied Sciences
A. Reis, D. Paulino, H. Paredes, I. Barroso, M.J. Monteiro, V. Rodgrigues, J. Barroso	Using intelligent personal assistants to assist the elderlies An evaluation of Amazon Alexa, Google Assistant, Microsoft Cortana, and Apple Siri	2018	Conference paper	2018 2nd International Conference on Technology and Innovation in Sports, Health and Wellbeing (TISHW)

		Intervention	
**Study objective**	**Category**	**Sub-category (Based on WHO categories of interventions for clients[1])**	**Description**
Conduct a preliminary test of a HPV vaccine-centric counseling conversational agent to help assess the idea of utilizing an automated conversational agent for HPV counseling in clinical environments	Support	Targeted health information based on health status	Automated counseling on HPV vaccination
Propose the possibility of automated discourse that could mimic patient-provider dialogue and provide consistent responses	Support	Targeted health information based on health status	Automated counseling on HPV vaccination
compare voice-activated internet searches by smartphone (two digital assistants) with laptop ones for information and advice related to smoking cessation	Support	Look-up of health information	Providing information or advice on smoking cessation.
Overcome lack of effectiveness of self-management mobile apps in elderly with a Google Home assistant application	Monitoring and Support	Look-up of health information<br>Active data capture/documentation<br>Targeted alerts and reminders	healthy coping and monitoring surveys,<br>medication reminders, information on activity and nutrition
Feasibility evaluation of whether the system could identify with high accuracy the information the patient provides in the interview (compared to nurse practitioners)	Monitoring	Active data capture/documentation	conducting self-care checkup via health monitoring interviews with chronic heart failure patients
Describes the concept and implementation of an interactive storytelling application for conversational-voice interfaces and reports the preliminary results of a pilot test	Support	Targeted health information based on health status	Provide people with intellectual disabilities with a playful, accessible, and cost-effective training platform to improve their social interaction abilities.
Detailing the realization of the concept of a conversational agent for the remote monitoring of audio and conversation dialogues in the form of a smartphone application	Monitoring	Self monitoring of health or diagnostic data	Remote monitoring of audio and conversation dialogues
Preliminary evaluation of a knowledge-enables personalized conversational agent to assist pediatric asthmatic patients	Monitoring and Support	Look-up of health information<br>Active data capture/documentation<br>Self monitoring of health or diagnostic data<br>Targeted alerts and reminders	Monitors asthma symptoms and checks both co-occurrence of factors potentially triggering asthma symptoms to then deliver warnings whenever the factors enter the unhealthy range
To design, develop and assess the usability of the concept of CARMIE as a virtual medication advisor	Monitoring and Support	Look-up of health information<br>Active data capture/documentation<br>Targeted health information based on health status	1) Deliver information and knowledge-based advice by providing assistance on posology, interactions, indications, and adverse reactions<br>2) assesses symptoms in case of out-of-prescription medicine inteka intention, and generates a medical report for the registered healthcare staff<br>3) To motivate the user<br>through interactive dialogue and cues, trying to increase medication adherence.
Introducing a self-conducted measurement via Smart Speaker for estimating the speech reception threshold	Monitoring	Active data capture/documentation	Conducting Matrix Sentence Test to deliver a speech reception threshold
Introduce a state-of-the-art virtual medical assistant, that interacts with the user in a spoken natural language, diagnoses a disease based on a user's chief complaint, and refers the user to a nearby appropriate medical specialist.	Monitoring and Support	Active data capture/documentation<br>Targeted health information based on health status	Listens to user's chief complaint and predicts a specific disease and instead of informing about the medical condition, it refers the user to a nearby appropriate medical specialist
Evaluate the feasibility of using<br>current consumer digital assistants to implement social interaction support routines	Support	Other (Task completion assistance)	Provide interactions scenarios with elderly people to promote social engagement, mobilize cognitive faculties and preserve mental health

[1]World Health Organization. Classification of digital health interventions v1.0. Sexual and reproductive health. 2018. URL: https://www.who.int/reproductivehealth/publications/mhealth/classification-digital-health-interventions/en/ [accessed 2021-03-12]

Voice-based Conversational Agent

Features	Description user interface	Description architecture	Name	Software implementation	Hardware implementation	Model of hardware implementation	Commercial availability
Delivery information about HPV vaccination; Delivery recommendations in case of parental resistance	Yes	Not applicable (Wizard of OZ)	-	iOS SDK	Tablet	-	Not available
-	No	Not applicable (Wizard of OZ)	Beverly	iOS SDK	Tablet	-	Not available
Delivery information about smoking cessation; Delivery advice on smoking cessation	No	No	Not applicable	Google Assistant and Siri	Smartphone	-	Available
Coping survey: PHQ-9 depression screening survey; Monitoring survey: Blood sugar values over time; Personalized advice for maintaining target glucose levels; Reminders for medication; Information on healthiness of food	No	Yes	Healthy Coping in Diabetes	Google Assistant	Smart speaker	Google Home	Not available
Collecting health information (e.g. weight, symptoms) through conversation and updated the user model	No	Yes	CARDIAC	CMUSphinx 3	-	-	Not available
Interactive storytelling game where user listens to a story and chooses from up to three predetermined story continuations varying in their thoughtfulness of the main character toward another character (a good choice, a negative choice and an adequate choice) and providing feedback depending on the adequateness of the choice made. User can choose the alternative either via word-based answer or by giving the associated letter (A/B/C).	No	No	-	Google Assistant; DialogFlow; Firebase	Smart speaker	Google Home	Not available
Demographics data collection; Call (randomly done by the chatbot between 8am and 8pm); Chat (initiated by the user); Audio data collection; Active speech analysis during the chat (how well vowels are articulated, vocabulary range, and duration of mid-sentence pauses.); User user consent for data logging after each interaction; User feedback on response consistency after each interaction	Yes	No	Harlie	Google Speech-to-Text; Google Text-to-Speech; random voice modulation; Artificial intelligence mark-up language (AIML)	Smartphone	-	Currently not available<br>(was in 2016 on Google Playstore)
- Deliver information about asthma zones, symptoms, triggers, medication usage and side-effects, and self-management skills<br>- Deliver informative rich media content (images and videos)<br>- Monitoring asthma symptoms via patient reports<br>- Monitoring of medication compliance history<br>- Monitoring of weather-related factors (e.g. pollen)<br>- Deliver personalized warnings based on monitoring co-occurrences	Yes	Yes	kBot	Google Android; Dialog Flow; Google Text-To-Speech API; Android SpeechRecognizer	Smartphone	-	Not available
Storage of medical prescriptions with posology; Delivery information a pharmacological property; Delivery suggestion in respose to a the patient asking for permission to take a medicine; In response of permission asking, it also assesses symptoms associated with the medicine and with deterioration of heart failure and provides suitable advice accordingly; Generatinof timestamped assessment answers as medical report available for sharing by email or text message	Yes	Yes	CARMIE	Android Speech; iSpeech Text to Speech (TTS) SDK	Smartphone	Samsung Galaxy Nexus	Not available
Conducting Matrix Sentence Test	No	Yes	Speech-controlled Automated Matrix Test (SAMT)	Amazon Alexa	Smart speaker	Alexa Echo Plus	Not available
Voice-based authentication for each utterance; Identification of a disease based on user's complaint; Deliver recommendations on specialists based on recognized disease; Conversation state tracking (where the conversation is with respect to the dialogue corpus); Continuous monitoring of system anomalies	Yes	Yes	MIRA	Android; Rasa framework	Smartphone	Samsung Galaxy S7 and iPhone 6s	Not available
Basic greeting: response to greeting; Email management: inform about mailbox status, write emails; Social media: inform about social events, publish birthday message on a social network; Social games: play game with user or with other online users through the VA	No	Yes	Not applicable	Google Assistant; Amazon Alexa; Apple Siri; Microsoft Cortana	-	-	Available

Health		Design			Participants						
Target illness	Target population	Type	Sub-type	N	Recruitment pool	Demographics	Age	Health status	Previous experience with technology	Previous experience with VCA[2]	Measures of technology acceptance
Cancers associated with Human Papillomavirus (HPV)	Parents of adolescents	Non-experimental	"Wizard of Oz Experiment"	16	"Participants recruited through flyers posted in the campus (probably of University of Texas)"	English-speaking, domain expertise, graduate degree, children of age <10	-	Healthy adults with at least one child under the age of 18	-	-	Validated questionnaire (PACV); Adapted questionnaire (Usability survey)
Cancers associated with Human Papillomavirus (HPV)	Parents of pediatric patients	Non-experimental	"Usability study with Wizard of Oz"	24	"Undergraduates of Texas A&M University (TAMU)"	Sex, Age (max, min), ethnicity, HPV vaccination reception	M=20	Healthy young adults between 18 and 16 years old	-	-	Validated questionnaire (SUS)
Cancers associated with smoking	Smokers	Non-experimental	-	No participants, 2 raters (authors)	Authors of the paper (2 raters)	Not applicable	Not applicable	Not applicable	Not applicable	Not applicable	Not applicable
Type-2 Diabetes Mellitus (T2DM)	T2DM elderly patients	Non-experimental	"Qualitative assessment of effectiveness and satisfaction"	10	"Willing elderly participants"	-	-	Healthy	-	-	Adapted questionnaire (effectiveness and satisfaction)
Heart Failure (HF)	Chronic heart failure patients	Non-experimental	"Feasibility evaluation"	14	"Chronic heart failure patients"	-	-	chronic heart failure patients	-	-	-
Intellectual disability	People with intellectual disability	Non-experimental	"User testing"	9	"Clients of a disability support service in Victoria, Australia"	Age max, min and SD	[19-30], SD=3.97	lifelong intellectual disability	Previous exposure to voice-based assistants was assessed but results not reported	-	-
Parkinson's Disease, Dementia, Autism	-	Non-experimental	"Focus group study"	33	"Elderly, members of community groups in Brisbane"	Sex, Age (max, min, mean)	[27, 87] M= 66.5	-	Smartphone ownership, Use competence in Androids, iPhones, tablets, laptops, desktop computers	-	Verbal feedback
Asthma	Asthma patients	Non-experimental	-	16	"Domain experts (clinicians) and non-domain experts (researchers)"	Domain expertise (clinicians vs researchers)	-	Healthy clinicians and researchers	-	-	Adapted questionnaire (Technology acceptance and quality); Validated questionnaire (SUS)
Heart Failure (HF)	HF elderly patients	Non-experimental	"Usability assessment"	11	"Native Portuguese adults"	Age (max, min)	[22-30]	Healthy (worked regularly with senior patients)	All were familiar with mHealth applications	-	Adapted questionnaire (SUS-based)
Hearing-impairment (HI)	HI listeners	Non-experimental	-	6	-	Age (max, min, mean, SD)	[20-28], M=24, SD=2.8	Normal-hearing	All had no experience with smart speakers	No	-
Diabetes (T1, T2, gestational) and Glaucoma	Glaucoma and diabetic patients	Non-experimental	"Case study"	33	"Members of Kyung Hee University, recruited via invitation email"	Sex, age max and min, professional domain, country	[18:43]	-	-	-	Validated questionnaire (UEQ)
Depression	Elderly	Non-experimental	-	-	"Selected and "tolerant" users"	-	-	-	-	-	-

[2]Voice-Based Conversational Agent

Evaluation

Constructs/Sub-categories of technology acceptance included	Measures of system accuracy	Behavioral measures	Measures of attitude towards the target health behavior	Technology acceptance
Usability; Free positive and negative comments	-	-	Parent Attitudes about Childhood Vaccines (PACV)	Good ease of use (5.4/7, SD=1.59); Acceptable expected capabilities (4.5/7, SD=1.46); Low efficiency (3.3/7, SD=1.85)
System Usability Survey (SUS); Speech User Interface; Service Quality (SUISQ)	-	-	Carolina HPV Immunization Attitude and Belief Scale (CHIAS)	SUS score higher score than industry standard of M=72/100, or C score (t(23)=1.627, p=0.059); SUS for no-HPV vaccine: M=80/100; SUS for vaccine group: M=77/100; SUS for do not know group: M=74/100. Note: SDs not provided.
Not applicable	Quality of the information and advice	Not applicable	Not applicable	Not applicable
User satisfaction; Preference Healthy Coping on Google Home over a smartphone	-	-	-	"VA more accepted than rejected in terms of user satisfaction"
-	Speech recognition accuracy: percentage of correct sentences (SC) recognized, word error rate (WER) performance.	-	-	-
-	User input recognition	Participants' progression through the game; Engagement<br>Time to respond; Points of difficulty; Points of dropout; Quality of responses	-	-
Initial impressions, difficulties encountered and practical applications	-	-	-	" Overall the impression from the first use of Harlie was positive, with participants also identifying technical problems (speed of processing), problematic conversational responses"
Naturalness; Information delivery; Interpretability; Technology acceptance; System Usability Scale (SUS)	-	-	-	Very good naturalness, information delivery, interpretability, technology acceptance (all 8.13≥);<br>Final SUS score clinicians: M=83.13/100,<br>Final SUS score researchers: M=82.81/100<br>(both SUS scores are equivalent to a 'very good' or 'B' rank score). Note: SDs not reported.
Usability; Coherence; Naturalness; Quality of information; HF-directed usability	-	-	-	Final SUS score: 88/100 (equivalent to a 'very good' or 'B' score). Note: SDs not reported.
-	Automatic speeach recognition (ASR) error rate; SRT measurement accuracy (compared to standard MST)	Verbal responses - non-matrix vocabulary; (NMV) words rate	-	-
Attractiveness; Perspicuity; Efficiency; Dependability; Stimulation; Novelty	Performance (accuracy, precision, sensitivity, specificity and f-measure); Task completion (success rate); Security breaches (VA accepting to talk with unidentified user)	-	-	Good user experience
-	Assessment via the criteria of Acknowledgment (of context); Engagement (coherent conversation); Effectiveness (full execution of interaction scenario); Usefulness (provision of useful and meaningful output); Follow-up (suggest and execute related activities)	-	-	-

Technology acceptance: details
Ease of use: M =5.4 (SD =1.59), Expected capabilities: M = 4.5 (SD =1.46), Efficiency: M = 3.3 (SD =1.85); Weak correlation Ease of use and efficiency (r = 0.34, 95% CI [−0.18, 0.72], p=0.197); Moderate correlation between ease of use and expected capabilities (r=0.63, 95% CI [0.08, 0.82], p=0.024) and between efficiency and expected capabilities (r=0.55, 95% CI [0.20, 0.86], p=0.007); Negative comments: the response time, the repetitiveness, the lack of visuals, the need to humanize the system, and the inability to answer all questions
SUS score higher score than industry standard of M=72/100 (t(23)=1.627, p=0.059); SUS score was higher among those that never had the vaccine (M=80/100) compared to those that did have the HPV vaccine (M=77/100) and those did not know if they had the HPV vaccine (M=74/100; Fisher Exact; Test of 12.42, p = 0.54); Correlation between SUS - SUISQ (r=0.486, p=0.016); Correlation User Goal Orientation and Verbosity (SUISQ) with SUS (r=0.508, p=0.005 and r=0.627, p=0.000, resp.); Correlation between Customer Service Behavior with SUS (r=0.438, p=0.014); Correlation SUISQ - Perceived Effectiveness (r= -.711, p=0.037); Verbosity (SUISQ) with Perceived Effectiveness (r= -0.764, p=0.014)
Not applicable
-
-
-
"Overall the impression from the first use of Harlie was positive, with participants also identifying technical problems (speed of processing), problematic conversational responses and potential future uses for Harlie [...] A frequent suggestion was providing company for people in residential aged care facilities."
Naturalness: clinicians M=8.25/10, researchers M=8.63/10; Information delivery: clinicians M=8.56/10, researchers M=8.44/10; Interpretability: clinicians M=8.25/10, researchers M=8.69/10; Technology acceptance: clinicians M=8.54/10, researchers M=8.63/10; System Usability Scale (SUS): clinicians M=83.13/100, researchers M=82.81/100. Note: SDs not reported.
The authors provided the average score (M) and the mean deviation (MD) of the questionnaire results. Usability: M=3, AD=0.87; Coherence: M=4, MD=0.63; Naturelness: M=3, AD=0.68; Quality of Information: M=3, MD=0.74; HR-directed Usability: M=3, MD=0.80. Note: no SD is provided.
-
User Experience (-3,+3): Attractiveness M=1.88/3; Perspicuity M=1.93/3; Efficiency M=1.88/3; Dependability M=1.70/3; Stimulation M=.90/3; and Novelty M=1.85/3. Note: mean values per construct are inferred manually; SD's are not provided.
-

Main findings

System accuracy	System accuracy: details
-	-
-	-
Google search performed best, followed by Google Assistant and Siri	Best advice: Google search: 83% (66/80); Google Assistant: 76% (61/80); Siri: 28% (22/80); NHS FAQ: Google search: 90% (32/35), Google Assistant: 79% (28/35), Siri: 49% (17/35); CDC questions: Google search: 79% (14/17), Google Assistant: 85% (15/17), Siri: 0% (0/17);<br>Best information: Pictures, locations, and other functionality questions: Google search: 75% (21/28), Google Assistant: 66% (19/28), Siri: 18% (5/28); Failed to provide any useful information: Google search: 9% (7/80), Google Assistant: 14% (12/80), Siri: 53% (42/80); First response was one or more advertisements: Google search: 21% (17/80), Google Assistant: 28% (22/80), Siri: 8% (6/80); Mean number of advertisements prior to a non-advertising response: Google search: 0.4 adverts, Google Assistant: 0.6 adverts, Siri: 0.3 adverts; Answer was from an expert source (grade A) (n = 52 questions from the NHS/CDC): Google search: 52% (27/52), Google Assistant: 49% (26/52), Siri: 24% (13/52); Answer was from a semi- expert source (grade B) (n = 52 questions from the NHS/CDC): Google search: 22% (12/52), Google Assistant: 20% (11/52), Siri: 13% (7/52); Answer was from a non-expert source (grade C) (n = 52 questions from the NHS/CDC): Google search: 22% (12/52), Google Assistant: 24% (13/52), Siri: 13% (7/52)
-	-
Sentence correct performance: ~50% in practice phase , 70% in interview; WER: ~40% in practice phase, ~20% in interview	-
Mediocre recognition of both A/B/C and word-based responses; A/B/C performs slightly better	43% recognition of A/B/C responses; 41% recognition of word-based responses
-	-
-	-
-	-
ASR error rate: Average score insertion<br>rate is (6.0 ± 2.3)%, Average score deletion<br>of (3.7 ± 1.0)%; SRT measurement accuracy: significant bias of 0.6 dB	ASR error rate: Average score insertion<br>rate is (6.0 ± 2.3)%, Average score deletion<br>of (3.7 ± 1.0)%; SRT measurement accuracy: significant bias of 0.6 dB; SRT bias: paired-sample t-test,<br>p = 0.002, M = −7.4±0.9 dB SNR with smart speaker, M = −8.0 ± 0.9 dB SNR with the starndard MST)
High accuracy, precision, sensitivity, and F-score; High success rate; No security breach	Performance: Average accuracy 89.8%; average precision 90%, average sensitivity: 89.9%, average specificity: 94.9%, F-score: 89.8%; Success rate: Expected agreement P(E) =0.334, Actual agreement P(A) = 0.898, Kappa Coefficient k=0.848 (i.e. Almost perfect); Security: no frequency reported
Basic greeting: good performance in all IPAs; Email: Heterogeneous results between IPAs, with Apple Siri performing best; Social network: good performance for all IPAs but Google Assistant; Social game: good performance for all IPAs but Siri	Basic greeting: all IPAs could acquire and maintain some context information from the user like the location but these services couldn´t provide any suggestion to the user, which would be a good point to continue the conversation as evaluated in the follow-up parameter; Email: some IPAs like Google Assistant and Microsoft Cortana only could write an email, Amazon Alexa could read and reply, while the Apple Siri could read and write an email. The follow-up parameter in this activity obtained bad results in every IPA with the highlight went to Amazon Alexa for suggesting the user to reply after reading an email to the sender, with predefined messages; Social network: all IPAs could let the user to manage their calendar and, except for Google Assistant, the possibility to send or read a message in a social network like Twitter or Facebook. The highlight in this activity went to the Amazon Alexa that allows the user to check the birthday calendars of their friends, this could be useful to letting the elderly people upkeep the birthdays dates of their relative ones. The follow-up in this activity also obtained bad results, with the IPA not suggesting anything to the user; Social game: present in almost all IPAs except the Apple Siri. The social games presented were mostly in quizzes format. The context data acquire from the IPA were practically none, but the IPA frequently had more initiative to follow-up in their dialog with the user suggesting some clues to help the user or giving other related games to play.

Behavior	Behavior: details
-	-
-	-
Not applicable	Not applicable
-	-
-	-
Participants performed relatively poorly and mostly used the A/B/C responses	Time for completion: M=3:22min, SD=1min; Quality of response: 3/9 made only good choices, 2/9 made one adequate choice, 4/9 made one negative choice, 7/9 made choices with A/B/C instead of word-based responses, 1/9 alternated between input types, 1/9 made choices with word-based responses; Engagement: 2/9 replayed the story, with 1/9 changed from A/B/C to word-based input, no participant gave the same A/B/C input consistently; Points of difficulty: No signs of frustration upon recognition errors
-	-
-	-
-	-
10.8% of the words contained in the participant's responses were irrelevant to the test sentence (e.g. "I understood [test sentence]")	Participants responded with 10.8% of non-vocabulary words (i.e. words that were not pasr ot the test sentence, e.g. "I understood [test sentence]")
-	-
-	-

Attitude towards health behavior	Attitude towards health behavior: details
PACV: all participants were categorized as non-hesitant	-
CHIAS: significant correlation with verbosity (SUISQ); In general, higher CHIAS compared to previous research	Correlation Verbosity (SUISQ) with Perceived Uncertainty (CHIAS; r= -0.874, p=0.05); Correlation SUISQ with Perceived Barriers and Perceived Effectiveness (r= -0.639, p=0.044 and r= -0.655, p=0.039, resp.); Differences between samples from Kamimura et al. and Hanson et al. and study sample: higher scores in CHIAS in the current study with respect to Perceived Barriers (p=.09), Risk Denial (p=0), Perceived Harms (p=0) and Perceived Uncertainty (p=0), but not with respect to Perceived Effectiveness (p=.26)
Not applicable	Not applicable
-	-
-	-
-	-
-	-
-	-
-	-
-	-
-	-
-	-

Additional information

Discussed limitations	Funding source(s)	Conflict of interests	Region	Continent
Wizard of Oz methodology influenced the perception of efficiency	UTHealth Innovation for Cancer Prevention Research Training Program; National Library of Medicine of the National Institutes of Health; National Institute of Allergy and Infectious Diseases of the National Institutes of Health	-	Texas, USA	NA
No control group; Small N; Not clear why so many participants did not know whether they received a HPV vaccine before; Sample differing from target population of parents of pediatric patients	UTHealth Innovation for Cancer Prevention Research Training Program; National Library of Medicine of the National Institutes of Health; National Institute of Allergy and Infectious Diseases of the National Institutes of Health	-	Texas, USA	NA
Results may be superior to reality as they used precise terms, i.e., no slang or colloquialisms	Self-funded by the authors	-	New Zeeland	OC
-	National Science Foundation (NSF); MU Interdisciplinary Innovations Fund	-	Cincinnati, USA	NA
-	Robert Wood Johnson Foundation NIH/NHLBI; Office of Naval Research	-	-	NA
-	Telematics Trust Fund, Victoria, Australia	-	Australia	OC
-	-	-	Australia	OC
-	National Institutes of Health (NICHD/NIH)	-	Ohio, USA	NA
CARMIE lacks in flexibility, mainly due to restricted; phrasings and a fixed output vocabulary; Study not conducted with the final target age group.; CARMIE is developed to be used in European Portuguese, a language still immature in what concerns natural language research, resources and tools, especially NLG and virtual intelligent assistants; Potential barrier of privacy concerns to the acceptance of this type of personal health application.	-	-	Portugal	EU
Tested in one specific condition (room, position); SRT is a value interpretable by experts only	Deutsche Forschungsgemeinschaft (DFG, German Research Foundation)	-	Germany	EU
-	Ministry of Science and Information and Communications Technology	The authors declare no conflict of interest.	South Korea	AS
Some results were ambiguous, as it was not possible to say IPAs could not execute the interactions but it was not suitable for an elderly user	European Regional Development Fund (ERDF)	-	Portugal	EU
