# Supplementary material for: Voice-Based Conversational Agents for the Prevention and Management of Chronic and Mental Health Conditions: Systematic Literature Review
Source: J Med Internet Res. 2021 Mar 29;23(3):e25933. doi: 10.2196/25933 (PMC8042539; doi:10.2196/25933)
Supplement: Multimedia Appendix 4 [file jmir_v23i3e25933_app4.pdf]

Multimedia Appendix: Risk-of-bias assessment (based upon the CONSORT checklist [1] and adapted from [2])

This is a Multimedia Appendix to a full manuscript published in the J Med Internet Res. For full copyright and citation information see http://dx.doi.org/10.2196/jmir.25933.

	Allen et al. 2009	Amith et al. 2019	Amith et al. 2020	Boyd and Wilson 2018	Ooster et al. 2019	Lobo et al. 2017	Cheng et al. 2019	Kadariya et al. 2019	Ireland et al. 2016	Rehman et al. 2020	Greuter and Balandin 2019	Reis et al. 2018
**1. Title and Abstract**<br>a. identification as a randomised trial in title<br>b. structured summary	0.25	0.25	0.25	0.5	0.25	0.25	0.25	0.25	0.25	0.25	0.25	0.25
**2. Introduction**<br>a. scientific background/ rationale<br>b. specific objectives/ hypotheses	0.75	1	1	1	1	1	1	1	1	1	1	1
**Methods**												
3. Trial design<br>*a. description of trial design*	1	1	1	1	1	1	0.25	1	0.25	1	0.5	1
**4. Participants**<br>a. eligibility criteria<br>b. settings and locations of data collection	0.25	0.75	1	0	0.75	0.5	0	0	0.25	0.5	0.75	0
**5. Interventions**<br>Descriptions with sufficient details to allow replication	0.25	0.5	0.5	0.5	0.5	0.5	0.25	0.5	0.5	0.5	0.5	0.25
6. Outcomes<br>*a. pre-specified primary and secondary outcome measures*	0.25	0.5	1	1	1	1	0.25	1	0.25	0.5	0.5	1
7. Sample size<br>*a. how sample size was determined*	0	0	0	0	0	0	0	0	0	0.25	0	0
**8. Randomisation - sequence generation**<br>a. method used<br>b. type of randomisation including details of any restriction	0	0	0	0	0	0	0	0	0	0	0	0
**9. Allocation concealment mechanism**<br>Implementation of the random allocation sequence, including concealment	0	0	0	0	0	0	0	0	0	0	0	0
**10. Implementation**<br>Who generated the random allocation sequence, who enrolled participants, and who assigned participants	0	0	0	0	0	0	0	0	0	0	0	0
**11. Blinding**<br>a. if done, who was blinded and how<br>b. if relevant, similarity of interventions	0	0	0	0	0	0	0	0	0	0	0	0
12. Statistical methods<br>*Statistical methods used for (primary and secondary) outcomes*	0	0.5	0.25	1	0	0.25	0	1	0.25	1	0	0.25
**Results**												
**13. Participants flow**<br>a. numbers of participants randomised, receiving treatment, and analysed<br>b. losses and exclusions, with reasons	0	0.75	0.5	0	0	0	0	0	0	0	0	0
14. Recruitement<br>*a. dates of recruitement and follow-up*	0	0.25	0.75	0.5	0.25	0	0	0	0	0.25	0	0
15. Baseline data<br>*a. baseline demographic and clinical characteristics*<br>*b. systematic table of characteristics for each group*	0	0.5	0.5	0	0.25	0.25	0	0	0.5	0.5	0.5	0
**16. Numbers analysed**<br>For each group, number of participants included in each analysis	0.5	0.5	0.5	0	0.5	0.5	0.5	1	0.5	0.5	0.5	0
17. Outcomes and estimation<br>*a. provides the estimated effect size*<br>*b. provides precision*	0.5	0.75	0.5	0.5	0.5	0.5	0.25	0.25	0.5	0.5	0.5	0.25
**18. Ancilliary analyses**<br>Results of any other analyses performed, distinguishing pre-specified from exploratory	0	0	0	0	0	0	0	0.5	0	0	0	0
**19. Harms**<br>Harms or unintended effects in each group	0	0	0	0	0	0	0	0	0	0	0	0
**Discussion**												
**20. Limitations**<br>Trial limitations/bias/ multiplicity of analyses	0	0.25	1	1	1	1	0	0	0	0.25	0	0
**21. Generalisability**<br>Generalisability (external validity, applicability) of findings	0	0	1	0.25	1	0.25	0	0	0.25	0.25	0	0
**22. Interpretation**<br>Consistent with results and balanced	0.5	0.25	0.5	0.5	1	1	0.25	0.25	0.25	0.5	0.25	0.25
**Other information**												
**23. Registration**<br>Registration number and name of registry	0.5	0	0	0	0	0	0	0.5	0	0.5	0	0.5
**24. Protocol**<br>Where the full trial protocol can be accessed	0	0	0	0	0	0	0	0	0	0	0	0
**25. Funding**<br>Sources of funding/ role of funders	1	1	1	1	1	0	1	1	0	1	1	1
	5	8.75	11.25	7.25	10	8	4	5	10.25	5.5	5.25	1
**Numbers of criteria satisfied**												

References:

1. Schulz KF, Altman DG, Moher D, CONSORT Group. CONSORT 2010 statement: updated guidelines for reporting parallel group randomised trials. PLoS Med 2010 Mar 24;7(3). doi: 10.1371/journal.pmed.1000251. Medline:20352064
2. Maher CA, Lewis LK, Ferrar K, Marshall S, Bourdeaudhuij ID, Vandelanotte C. Are health behavior change interventions that use online social networks effective? A systematic review. J Med Internet Res 2014 Feb 14;16(2):e40. doi: 10.2196/jmir.2952. Medline: 24550083
