# Supplementary material for: Voice-Based Conversational Agents for the Prevention and Management of Chronic and Mental Health Conditions: Systematic Literature Review
Source: J Med Internet Res. 2021 Mar 29;23(3):e25933. doi: 10.2196/25933 (PMC8042539; doi:10.2196/25933)
Supplement: Multimedia Appendix 5 [file jmir_v23i3e25933_app5.pdf]

Multimedia Appendix 3: Main characteristics of the included studies

This is a Multimedia Appendix to a full manuscript published in the J Med Internet Res. For full copyright and citation information see http://dx.doi.org/10.2196/jmir.25933.

Characteristics and main findings of the included studies

Study ID	Year of publication	Paper type	Study aim category
Amith et al (2019)	2019	Journal paper	Development and acceptance evaluation
Amith et al (2020)	2020	Congress paper	Development and acceptance evaluation
Boyd and Wilson (2018)	2018	Journal paper	Criteria-based performance evaluation of commercial conversational agent
Cheng et al (2019)	2019	Conference paper	Development and acceptance evaluation
Galescu et al (2009)	2009	Conference paper	Development and performance evaluation
Greuter and Balandin (2019)	2019	Conference paper	Development and performance evaluation
Ireland et al (2016)	2016	Journal paper	Development and acceptance evaluation
Kadariya et al. (2019)	2019	Conference paper	Development and acceptance evaluation
Lobo et al (2017)	2017	Journal paper	Development and acceptance evaluation
Ooster et al (2019)	2019	Conference paper	Development and performance evaluation
Rehman et al (2020)	2020	Journal paper	Development and performance & acceptance evaluation
Reis et al (2018)	2018	Conference paper	Criteria-based performance evaluation of commercial conversational agent

	Study aim details	Study Design	Design sub-type (verbatim)
Amith et al (2019)	Assess the idea of utilizing an automated conversational agent for HPV vaccine counseling in clinical environments	Nonexperimental	Wizard of Oz Experiment
Amith et al (2020)	Assess the idea of utilizing of automated conversational agent mimicking patient-provider dialogue and provide consistent responses around HPV vaccine	Nonexperimental	Wizard of Oz Experiment
Boyd and Wilson (2018)	Comparison voice-activated internet search via smartphone (two digital assistants) with standard laptop search for information and advice related to smoking cessation	Nonexperimental	-
Cheng et al (2019)	Using Google Home to host verbal and visual data communication and reduce the burden diabetes monitoring and self-management in elderlies	Nonexperimental	Qualitative assessment of effectiveness and satisfaction
Galescu et al (2009)	Evaluating if the conversational agent could identify relevant health-information via automated interview for self-conducted check-up (compared to nurse practitioners)	Nonexperimental	Feasibility evaluation
Greuter and Balandin (2019)	Implementing an interactive storytelling application to train social skills, requiring the user to select the best response (by saing "A", "B", or "C", or by giving a word-based response) to potentially challenging social interactions.	Nonexperimental	Pilot study
Ireland et al (2016)	Implementatoin of a voice assistant for remote monitoring of audio and conversation dialogues to understand the impact of health conditions on communication	Nonexperimental	Focus group study
Kadariya et al. (2019)	Preliminary evaluation of a knowledge-enabled personalized conversational agent to assist pediatric patients with asthma control	Nonexperimental	-
Lobo et al (2017)	To design, develop and assess the usability of the concept of CARMIE as a virtual medication advisor	Nonexperimental	Usability study
Ooster et al (2019)	Adaptation of the matrix sentence tests to a smart speaker for speech reception threshold estimation	Nonexperimental	-
Rehman et al (2020)	Introduction of a virtual medical assistant, that interacts with the user in a spoken natural language, indentfies the user based on their voice, diagnoses a disease based on a user's chief complaint, and refers the user to a nearby appropriate medical specialist.	Nonexperimental	Case study
Reis et al (2018)	Evaluate the feasibility of using<br>current consumer digital assistants to support social interaction in elderlies, via conversational routines, such as basic greetings, email management, social network management, and playing social games	Nonexperimental	-

	Main accuracy findings	Main technology acceptance findings
Amith et al (2019)	Not applicable	Good ease of use (M=5.4/7, SD=1.59)<br>Acceptable expected capabilities (M=4.5/7, SD=1.46)<br>Low efficiency (M=3.3/7, SD=1.85)<br>Good usability (SUS score sign. higher than industry standard of M=72/100, 'C' rank score, no SD provided)<br>SUS for no-HPV vaccine: M=80/100, no SD provided<br>SUS for vaccine group: M=77/100, no SD provided<br>SUS for do not know group: M=74/100, no SD provided
Amith et al (2020)	Not applicable	Medium speech user interface quality (SUISQ score M=4.29/7, SD=0.75)
Boyd and Wilson (2018)	Google search performed best, followed by Google Assistant and Siri	Not applicable
Cheng et al (2019)	Not applicable	VA more accepted than rejected in terms of user satisfaction
Galescu et al (2009)	Good speech recognition performance (70%)<br>Acceptable error rate (20%)	Not applicable
Greuter and Balandin (2019)	Mediocre speech recognition accuracy in both A/B/C (43%) and word-based (41%) responses;<br>A/B/C performs slightly better	Not applicable
Ireland et al (2016)	Not applicable	General positive assessment, except for the slowness of processing
Kadariya et al. (2019)	Not applicable	For both clinicians and researchers:<br>Very good naturaleness (M=8.25/10 and M=8.62/10, resp.), information delivery (M=8.56/10 and M=8.44/10, resp.), interpretability (M=8.25/10 and M=8.69/10, resp.), technology acceptance (M=8.54/10 and M=8.63/10, resp, no SD provided)<br>Very good usability (SUS score, clinicians: M=83.13/100, researchers: M=82.81/100, both 'B' rank score, no SD provided)
Lobo et al (2017)	Not applicable	Very good usability (SUS score M = 88/100, 'B' rank score, no SD provided)
Ooster et al (2019)	Good speech recognition performance (false positive 6%, false negative 3.7%);<br>Significant underestimation of hearing threashold compared to standard (i.e. overestimation hearing abilities)	Not applicable
Rehman et al (2020)	Very good speech recognition accuracy, precision, sensitivity, and F-score (all ≥89%);<br>High success rate (84.8%)<br>No security breach	Positive user experience (Attractiveness: M=1.88/3, Perspicuity: M=1.93/3, Efficiency: M=1.88/3, Dependability: M=1.70/3, Stimulation: M=1.90/3, Novelty: M=1.85/3, scores manually inferred by the authors of this review, no SD provided)
Reis et al (2018)	1. Basic greeting: good performance in all IPAs;<br>2. Email: Heterogeneous results betweeen IPAs, with Apple Siri performing best;<br>3. Social network: good performance for all IPAs but Google Assistant;<br>4. Social game: goog performance for all IPAs but Siri	Not applicable

NB: SUS = System Usability Survey

Methodology of the included studies

Study ID	Behavioral measures	System measures	Acceptance measures
Amith et al (2019)	-	-	Adapted questionnaire on usability<br>Qualitative feedback
Amith et al (2020)	-	-	Validated questionnaires (SUS, SUISQ)
Boyd and Wilson (2018)	-	Classification of quality of the information and advice based on source of information (i.e. sites of health agencies, sites with "some expertise", sites with subjective content)	-
Cheng et al (2019)	-	-	Adapted questionnaire on effectiveness and satisfaction and on preference of Healthy Coping on Google Home over a smartphone
Galescu et al (2009)	-	Speech recognition percentage<br>Speech recognition error rate	-
Greuter and Balandin (2019)	Engagement<br>Performance (task completion, time to respond, points of difficulty, points of dropout, quality of responses)	Speech recognition percentage	-
Ireland et al (2016)	-	-	Qualitative feedback
Kadariya et al. (2019)	-	-	Adapted questionnaire on technology acceptance and quality (Naturalness, Information delivery, Interpretability)<br>Validated questionnaire (SUS)
Lobo et al (2017)	-	-	Adapted SUS-based questionnaire (Usability, Coherence, Naturalness, Quality of information, Heart-failure-directed usability)
Ooster et al (2019)	Verbal responses (percentage of non-matrix vocabulary words)	Speech recognition error rate<br>Speech reception threshold measurement accuracy (compared to standard Matrix Sentence Test)	-
Rehman et al (2020)	-	Speech recognition accuracy, precision, sensitivity, specificity and f-measure<br>Task completion (success rate)<br>Prevention from security breaches (conversational agent accepting to talk with unauthenticated user)	Validated questionnaire (UEQ)
Reis et al (2018)	Not applicable	Pre-defined criteria:<br>Acknowledgment (of context)<br>Engagement (coherent conversation)<br>Effectiveness (full execution of interaction scenario)<br>Usefulness (provision of usedul and meaninful output)<br>Follow-up (suggest and execute related activities)	Not applicable

NB: SUS = System Usability Survey, SUISQ = Speech User Interface-Service Quality, UEQ = User Experience Questionnaire

	Measures of attitude towards the target health behavior	Reported experience with technology
Amith et al (2019)	Validated questionnaire (Parent Attitudes about Childhood Vaccines, PACV)	-
Amith et al (2020)	Validated questionnaire (Carolina HPV Immunization Attitude and Belief Scale, CHIAS)	-
Boyd and Wilson (2018)	Not applicable	Not applicable
Cheng et al (2019)	Not applicable	-
Galescu et al (2009)	-	-
Greuter and Balandin (2019)	-	Previous exposure to voice-based assistants was assessed but results not reported
Ireland et al (2016)	-	Smartphone ownership, Use competence in Androids, iphones, tablets, laptops, desktop computers
Kadariya et al. (2019)	-	-
Lobo et al (2017)	-	All were familiar with mHealth applications
Ooster et al (2019)	-	All had no experience with smart speakers
Rehman et al (2020)	-	-
Reis et al (2018)	Not applicable	Not applicable

Health Characteristics

Study ID	Target chronic condition	Target population	Tested sample (n)
Amith et al (2019)	Cancers associated with Human Papillomavirus	Parents of adolescents	Healthy adults with at least one child under age of 18 (n=16)
Amith et al (2020)	Cancers associated with Human Papillomavirus	Parents of pediatric patients	Healthy young adults between 18 and 16 years old (n=24)
Boyd and Wilson (2018)	Cancers associated with smoking	Smokers	Authors themselves (n=2)
Cheng et al (2019)	Diabetes (T2)	Elderly patients	Elderly (n=10)
Galescu et al (2009)	Heart Failure	Chronic heart failure patients	Chronic heart failure patients (n=14)
Greuter and Balandin (2019)	Intellectual disability	People with intellectual disability	Adults with lifelong intellectual disability (n=9)
Ireland et al (2016)	Parkinson Disease, Dementia, Autism	-	Adults recruited on campus (n=33)
Kadariya et al. (2019)	Asthma	Asthma patients	Clinicians and Researchers (n=16)
Lobo et al (2017)	Heart Failure	Elderly patients	Healthy adults working regularly with senior patients (n=11)
Ooster et al (2019)	Hearing-impairment	Hearing-impaired listeners	Normal-hearing (n=6)
Rehman et al (2020)	Diabetes (T1, T2, gestational) and Glaucoma	Glaucoma and diabetic patients	Adults affiliated to the university (n=33)
Reis et al (2018)	Depression	Elderly	-

Characteristics of Voice-based Conversational Agents

Study ID	Name	Description user interface	Description architecture
Amith et al (2019)	-	Yes	Not applicable (Wizard of OZ)
Amith et al (2020)	Beverly	No	Not applicable (Wizard of OZ)
Boyd and Wilson (2018)	Commercial (Google, Siri)	No	No
Cheng et al (2019)	Healthy Coping in Diabetes	No	Yes
Galescu et al (2009)	Cardiac	No	Yes
Greuter and Balandin (2019)	-	No	No
Ireland et al (2016)	Harlie	Yes	No
Kadariya et al. (2019)	kBot	Yes	Yes
Lobo et al (2017)	Carmie	Yes	Yes
Ooster et al (2019)	Samt	No	Yes
Rehman et al (2020)	Mira	Yes	Yes
Reis et al (2018)	Commercial (Alexa, Cortana, Google, Siri)	No	Yes

	Device	Commercial availability
Amith et al (2019)	Tablet	Not available
Amith et al (2020)	Tablet	Not available
Boyd and Wilson (2018)	Smartphone	Available
Cheng et al (2019)	Smart speaker	Not available
Galescu et al (2009)	-	Not available
Greuter and Balandin (2019)	Smart speaker	Not available
Ireland et al (2016)	Smartphone	Not available; (was available in 2016 on Google Playstore)
Kadariya et al. (2019)	Smartphone	Not available
Lobo et al (2017)	Smartphone	Not available
Ooster et al (2019)	Smart speaker	Not available
Rehman et al (2020)	Smartphone	Not available
Reis et al (2018)	-	Available

Characteristics of Voice-Based Interventions

Study ID	Intervention category	Intervention[1]
Amith et al (2019)	Support	Targeted health information based on health status
Amith et al (2020)	Support	Targeted health information based on health status
Boyd and Wilson (2018)	Support	Look-up of health information
Cheng et al (2019)	Monitoring	Active data capture/documentation
	Support	Look-up of health information; Targeted alerts and reminders
Galescu et al (2009)	Monitoring	Active data capture/documentation
Greuter and Balandin (2019)	Support	Targeted health information based on health status
Ireland et al (2016)	Monitoring	Self monitoring of health or diagnostic data
Kadariya et al. (2019)	Monitoring	Active data capture/documentation; Self monitoring of health or diagnostic data
	Support	Look-up of health information; Targeted alerts and reminders
Lobo et al (2017)	Monitoring	Active data capture/documentation
	Support	Look-up of health information; Targeted health information based on health status
Ooster et al (2019)	Monitoring	Active data capture/documentation
Rehman et al (2020)	Monitoring	Active data capture/documentation
	Support	Targeted health information based on health status
Reis et al (2018)	Support	Other: Task completion assistance

[1]Categorization of intervention based on the *Classification of digital health interventions v1. 0* from the World Health Organization [1].

	Intervention details
Amith et al (2019)	Delivery information about HPV vaccination and recommendations in case of parental resitance
Amith et al (2020)	Automated counseling on HPV vaccination
Boyd and Wilson (2018)	Providing information or advice on smoking cessation.
Cheng et al (2019)	Conducing PHQ-9 depression screening survey and monitoring survey on blood sugar values over time
	Advice to maintain glucose level, information on healthiness of food
Galescu et al (2009)	Collecting health information (e.g. weight, symptoms) through conversation and updated the user model
Greuter and Balandin (2019)	Interactive storytelling game where user listens to a story and chooses from up to three predetermined story continuations varying in their thoughtfullness of the main character toward another character; A good choice, a negative choice and an adequate choice) and providing feedback depending on the adequateness of the choice made. User can choose the alternative either via word-based answer or by giving the associated letter (A/B/C).
Ireland et al (2016)	Random calls by the conversational agent between 8am and 8pm and possibility for user-initiated call; Data collection on demographics, audio, user consent for data logging and user feedback on response consistency after each interaction; Active speech analysis during the conversation (how well vowels are articulated, vocabulary range, and duration of mid-sentence pauses.)
Kadariya et al. (2019)	Monitors asthma symptoms via patient reports and checks both co-occurence of factors potentially triggering asthma symptoms (medication compliance, weather-related factors) to then deliver warnings whenever the factors enter the unhealthy range
	Delivers information about asthma zones, symptoms, triggers, medication usage and side-effects, and self-management skills; Delivers informative rich media content (images and videos)
Lobo et al (2017)	Assesses symptoms in case of out-of-prescription medicine intake intention, and generates a medical report for the registered healthcare staff
	Deliver information and knowledge-based advice by providing assistance on posology, interactions, indications, and adverse reactions; To motivate the user through interactive dialogue and cues, to increase medication adherence
Ooster et al (2019)	Conducting Matrix Sentence Test to deliver a speech reception threshold
Rehman et al (2020)	Authenticates user via voice in each utterance; Performs continuous monitoring of conversation state (i.e. where the conversation is with respect to the dialogue corpus) and of possible system anomalies; Listens to user's chief complaint and predicts a specific disease
	Based on complains, refers the user to a nearby appropriate medical specialist
Reis et al (2018)	Provide 4 interactions scearios with elderly people to promote social engagement, mobilize cognitive faculties and preserve mental health. (1) Basic greeting: responds to greetings; (2) Email management: informs about emailbox status, write emails; (3) Social media: informs about social events, publish birthday message on a social network; (4) Social games: plays games with user or with other online users through the conversational agent

Reference:

1. World Health Organization. Classification of digital health interventions v1.0. Sexual and reproductive health. 2018. URL: https://www.who.int/reproductivehealth/publications/mhealth/classification-digital-health-interventions/en/ [accessed 2021-03-12]
